# Exostosis or osteochondroma

**DOI:** 10.11604/pamj.2024.49.107.18777

**Published:** 2024-12-03

**Authors:** Azhar Salim Mohamed, Mohamed Hachim

**Affiliations:** 1 Médecine Générale et Médecine du Travail, Centre de Santé des HLM, Dakar, Sénégal,; 2 Service de Chirurgie Orthopédique et Traumatologique du CHU Aristide Le Dantec de Dakar, Dakar, Sénégal

**Keywords:** Exostosis, complications, imaging, benign bone tumor

## Image in medicine

The osteochondroma or exostosis is the most common benign bone tumor. Exostosis may occur as solitary or multiple tumors, hereditary multiple exostoses. It is localized mostly in the long bones, first in a metaphyseal site then gradually diaphyso-metaphyseal by migration following the elongation of the distal portion of the bone. We report a case of a 24-year-old male who consulted at the HLM Health Center in Dakar, for pain and increased volume of the anterolateral aspect of the left knee that occurred 24 hours after a closed trauma. On examination, we noted lameness and painful swelling on palpation. Flexion and extension of the knee were preserved. The rest of the exam was normal. An X-ray of the affected knee was made and revealed a pedicled homogeneous bone excrescence with a cartilaginous matrix at the medial side of the distal metaphysis of the left femur. This bone excrescence was lined with a cortical continuity with the femoral bone without periosteal reaction or anomaly of soft parts. The patient was put on analgesic (paracetamol) and a non-steroidal anti-inflammatory drug (diclofenac) and then referred to a trauma-orthopedic department. Complications associated with exostosis include osseous deformity, fracture, vascular compromise, neurologic sequelae, and malignant transformation.

**Figure 1:**
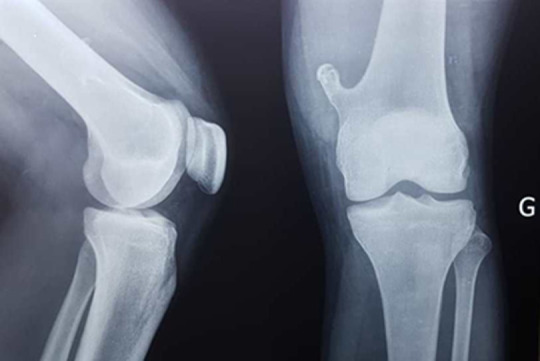
X-ray of the left knee in front and profile showing exostosis of the lower end of the femur

